# Isolation and Characterization of a Salt Inducible Promoter from *Chlorella vulgaris* PKVL7422

**DOI:** 10.4014/jmb.2304.04005

**Published:** 2023-04-28

**Authors:** Min-Jeong Kim, Su-Hyun Kim, Najib Abdellaoui, Tae-Jin Choi

**Affiliations:** 1 School of Marine and Fisheries Sciences, Pukyong National University, Busan 46241, Republic of Korea; 2 Department of Biological Sciences, Kongju National University, Kongju 32588, Republic of Korea

**Keywords:** *Chlorella vulgaris*, inducible promoter, microalgae, salt stress, transformation

## Abstract

*Chlorella* is a eukaryotic organism that can be used as an industrial host to produce recombinant proteins. In this study, a salt-inducible promoter (SIP) was isolated from the freshwater species *Chlorella vulgaris* PKVL7422 from the screening of genes that were upregulated after salt treatment. Several *cis*-acting elements, including stress response elements, were identified in the isolated SIP. Moreover, the Gaussia luciferase gene was cloned after the SIP and transformed into *C. vulgaris* to test the inducibility of this promoter. Reexamination of transcriptome of *C. vulgaris* revealed that genes involved in the synthesis of methyl jasmonic acid (MeJA), gibberellin (GA), and abscisic acid (ABA) were upregulated when *C. vulgaris* was treated with salt. Furthermore, the expression level of recombinant luciferase increased when the transformed *C. vulgaris* was treated with salt and MeJA, GA, and ABA. This study represents the first report of the *C. vulgaris* SIP and highlights how transformed microalgae could be used for robust expression of recombinant proteins.

## Introduction

Microalgae, the major primary producers on Earth, perform approximately 50% of global CO_2_ fixation [1]. They have been utilized in industries such as human and animal food production, nutrition, cosmetics, and pharmaceuticals [2]. Recent advances in biotechnology have enabled genetic manipulation of microalgae species to enhance the production of natural compounds, a process known as metabolic engineering [3]. For instance, Zhang *et al*. [4] increased the lipid content of *Chlorella ellipsoidea* from 46.4% to 52.9% via transformation and expression of a soybean transcription factor, GmDof4. Similarly, the overexpression of phytoene desaturase in *C. zofingiensis* resulted in a 32.1% increase in total carotenoids and a 54.1% increase in astaxanthin [5].

In addition to their genetic manipulation capacity, microalgae have many desirable features that could support their utilization as bioreactors to produce recombinant proteins (*e.g.*, enzymes, vaccines, and antibodies), which have considerable value in industrial and pharmaceutical applications [2]. Although the growth of microalgae is slower than that of bacteria and yeast used as host for recombinant protein production, their growth with a doubling time of 24 h is faster than that of plants, insect cells and mammalian cells used for the production of many eukaryotic [6-9]. Additionally, many heterotrophic microalgae can be cultured in enclosed photobioreactors or fermenters to reduce the chance of genetically modified organisms from escaping or contaminating the culture [10]. Microalgae used as heterologous expression systems are eukaryotes; thus, they have post-translational modification systems (*e.g.*, disulfide bond formation, phosphorylation, and glycosylation), which are necessary for the function of some recombinant proteins of eukaryotic origin [11]. Compared with mammalian systems, which are the primary bioreactors for recombinant protein production, the cost of microalgae cultures is significantly lower. Furthermore, the unicellular nature of most microalgae can simplify downstream processing [12]. Despite the numerous advantages of microalgae expression systems, their development remains in its infancy; there are several limitations, including the lack of an efficient transformation system, lack of reliable low-cost selection markers, poor stability of introduced foreign DNA in the absence of selection pressure, and low expression of recombinant proteins [11, 12].

Various factors can influence the expression levels of recombinant proteins, including *cis*-acting promoters, codon bias, *trans*-acting regulatory factors, gene silencing, and host protease degradation. Among these factors, promoters that determine the transcriptional activities of introduced genes are a major concern in microalgae transformation systems. The cauliflower mosaic virus 35S promoter (CaMV 35S), which strongly induces expression in most dicotyledonous and some monocotyledonous plants, can also be used in microalgae transformation systems [2, 13]. Strong constitutive endogenous promoters such as rubisco small subunit 2 (RbcS2) and *Chlamydomonas* photosystem I complex protein (psaD) can also be used for recombinant protein production [14], along with inducible promoters; the use of both types of promoters can support a two-phase production system in which normal culture conditions can be used for optimal microalgal growth, and other conditions can be used for enhanced expression of recombinant proteins to maximize yield. For example, the *Chlamydomonas* heat shock protein 70A promoter demonstrated enhanced production of recombinant proteins under heat stress when used alone or in combination with other promoters [15]. The nitrate reductase (NR) inducible promoter has also been used in microalgae transformation systems because it is repressed in the presence of ammonium and induced when ammonium is replaced with nitrate [16-17].

Salt stress can increase *Chlorella* biomass [18] and improve gene expression in plants (BY-2 cells and rice), bacteria (*Halomonas elongate*), and microalgae (*Chlamydomonas reinhardtii*) [19-21]. In a previous study, we investigated the effects of a high-salt environment on *Chlorella* and identified genes that were upregulated because of salt stress [22].

In the present study, we isolated the promoter regions of genes upregulated under salt stress, and then developed a high-level expression system that can be induced with salt treatment.

## Materials and Methods

### Selection of Upregulated Genes under Salt Condition

The salt experiment and transcriptome analysis were conducted as in the work of Abdellaoui *et al*. [22]. Briefly, *C. vulgaris* PKVL7422 (KCTC13361BP) was cultured in mBG11 medium [23] at 20°C with a light source of 52 μmol photons/m^2^/s^1^. Cultures were treated with 170 mM (1%) or 510 mM (3%) NaCl for 2 h and 4 h have described previous [22]. RNA was extracted from the concentrated cells and subjected to transcriptome analysis [22]. The expression levels of transcripts in treated groups were compared with the control group via RNA-Seq by Expectation-Maximization v1.2.15 based on Fragments Per Kilobase of transcript per Million (FPKM) values. Transcripts with zero FPKM were removed; the remaining FPKM values were log-transformed and normalized using a quantile normalization method [24]. Transcript expression was calculated based on the fold change between control and treatment groups, with a default cutoff of ± 2.

### qPCR of Gene 2 Transcripts after Salt Treatment

*C. vulgaris* PKVL7422 was cultured in mBG11 medium and treated with 170 mM (1%), 250 mM (1.5%), 340 mM (2%), or 510 mM (3%) NaCl for 30, 60, and 120 min. Total RNA extraction and cDNA synthesis were performed as described in the previous study [22]. qPCR was conducted using gene 2 primer sets, AccuPower 2X GreenStar™ qPCR Master Mix (Bioneer, Korea), and a QuantStudio™ 6 Flex Real-Time PCR System (Thermo Fisher, USA) (Table 1). The qPCR conditions were 95°C for 15 min followed by 40 cycles of denaturation at 94°C for 15 s, annealing at 55°C for 30 s, and extension at 70°C for 30 s. Expression levels were calculated using the 2-ΔΔct method [25].

### Cloning and Sequence Analysis of the Salt-Inducible Gene 2 Promoter

The gene 2 nucleotide sequence was obtained from previous transcriptome analysis [22], and the corresponding gene was identified by alignment with the genomic DNA sequence of *C. variabilis* hypothetical protein (GenBank Accession number XP_005848099.1). A DNA fragment 1,174 bp in length covering positions 14,822–15,996 of the above sequence containing the putative promoter region was amplified with SIP primers using the same PCR conditions described above for target gene detection (Table 1). The separated PCR product was purified from a 1%agarose gel using the GEL SV Kit (Gene All, Korea) and cloned into the pMD19 vector (Takara, Japan). After the cloned DNA had been sequenced, the sequence was analyzed to identify promoter elements using the PlantCARE database (http://bioinformatics.psb.ugent.be/webtools/plantcare/html/).

### Construction of the *Chlorella* Transformation Vector

A transformation vector for *Chlorella* was created, incorporating the SIP of gene 2 by modification of a previously developed vector, pCCVG which has two flanking sequences of 1,000bp originated from the NR gene of *C. vulgaris* PKVL7422 for the integration by double homologous recombination encompassing the cauliflower mosaic virus 35S promoter-Viral Hemorrhagic Septicemia Virus glycoprotein gene-transcription terminator from the RbcS2 gene of *Chlamydomonas reinhardtii* [23]. The modified Gaussia luciferase gene (GLuc) was synthesized by Bionics (Korea) based on the sequence of GenBank accession number AY015993.1 and codon optimization based on the *C. vulgaris* codon database (https://zendto.bioneer.co.kr/codon/index.py/), cloned into uP57-Amp vector. To create the vector pCCGL (*C. vulgaris* NR_CaMV 35S_GLuc_ vector), pCCVG and the GLuc gene were digested with BamHI and XhoI restriction enzymes, and the VHSV glycoprotein gene was replaced with the GLuc gene. The SIP-T vector was created by insertion of EcoRI at the 5’ end and BamHI at the 3’ end of the SIP. Using BamHI and EcoRI restriction enzymes, the CaMV 35S promoter in pCCGL and pSIP-T was replaced with SIP, resulting in the vector pCSGL (*C. vulgaris* NR_SIP_GLuc_vector) (Fig. S1).

### *Chlorella* Transformation

**Fig. 1:**
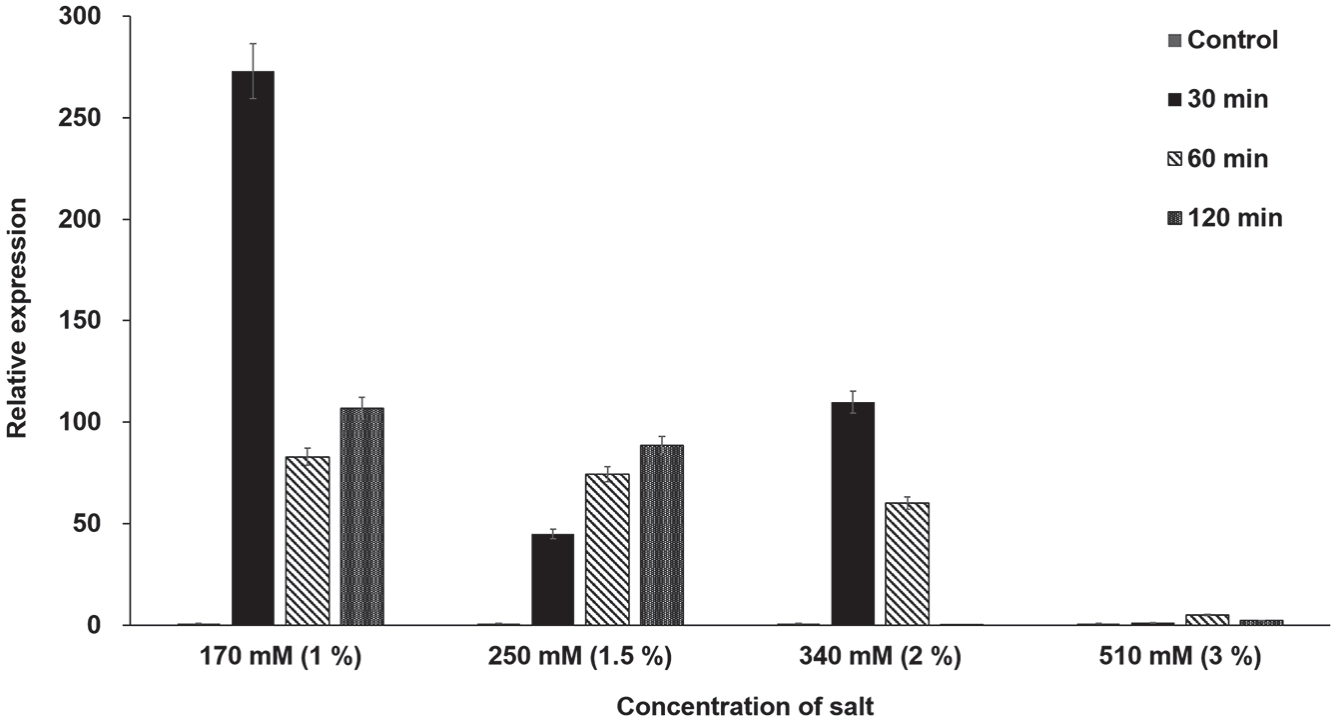
Effect of salt stress on gene 2 expression. qPCR was performed in replicates to determine the effect of salt stress on gene 2. The y-axis depicts expression levels relative to the level in untreated wild-type *C. vulgaris* PKVL7422.

To avoid integration of the ampicillin resistance gene from the pCSGL vectors into the *Chlorella* genome, a 5,013-bp-long DNA fragment containing the 5’ and 3’ NR gene flanking sequence was amplified using NR primers (Fig. 1, Table 1). PCR amplification was performed using the EzPCR LF 5x PCR Master mix (Elpis-Biotech, Korea) under the following conditions: pre-denaturation at 95°C for 3 min; 35 cycles of denaturation at 95°C for 20 s, annealing at 59°C for 20 s, and extension at 72°C for 4 min; and a final extension at 72°C for 10 min. The PCR product was isolated by electrophoresis on a 1% (w/v) agarose gel, then purified using the DOKDO-Prep™ Gel Extraction Kit (Elpis-Biotech). Two milliliters of a *C. vulgaris* PKVL7422 culture grown in BG11 medium to a density of 2 × 10^7^ cells/ml were harvested by centrifugation at 3,000 ×*g* for 30 min at 4°C. The resulting pellet was resuspended in 400 μl of osmosis buffer (200 mM D-sorbitol, 200 mM D-mannitol) and incubated for 1 h at room temperature. After centrifugation at 4,000 ×*g* for 10 min, the pellet was resuspended in 400 μl of electroporation buffer (500 mM NaCl, 200 mM D-mannitol, 200 mM D-sorbitol, 20 mM HEPES, 5 mM CaCl_2_, 5 mM KCl; pH 7.2); 4 μg of DNA fragment were added, and the mixture was incubated on ice for 10 min. The mixture was then transferred into 0.2-cm electroporation cuvettes; electroporation was conducted using the Gene Pulser II (Bio-Rad, USA) at 1.0 kV, 25 μF, and 400 Ω for 5 s. Treated cells were transferred to a 6-well plate containing 5 ml of BGNK broth [20] and stabilized in the dark at 20°C for 18 h, then plated on a BGNK agar plate containing 200 mM KClO_3_ to select transformed cells whose NR gene that reduce ClO_3_^-^ into toxic ClO_2_^-^ was replaced with the insert DNA, and could survive on KClO_3_ plate [23, 26-28]. Plates were incubated under continuous dark conditions at 20°C for 14 days.

### Selection and Confirmation of Transformed Cells

After 14 days of incubation, 9 colonies of transformed cells were randomly selected and used to inoculate 5 ml of BGNK broth, in accordance with the method of Kim *et al*. [20]; the broth cultures were grown for 7 days at 20°C under continuous cool fluorescent light of 52 μmol photons/m^2^/s. The 5-min plant DNA extraction kit (New England Biolabs, USA) was used to extract DNA in accordance with the manufacturer’s protocol, and the GLuc gene was amplified with GLuc primers (Table 1). The selected colonies were cultured and subjected to a luciferase assay, and the colony with the highest activity was chosen for further analysis.

### Luciferase Assay

Luciferase assays were performed to measure promoter activity. Transformed and wild-type *C. vulgaris* cells were inoculated at a density of 1.5 × 10^6^ cells/ml and incubated for 2 days. Then, ABA, MeJA and GA dissolved in DMSO was added to the final concentrations of 0 μM, 1 μM, 5 μM, 10 μM, 15 μM, 20 μM, 50 μM, and 100 μM. The culture was also treated with 0 mM, 100 mM, 150 mM, 200 mM, 250 mM, 300 mM, 350 mM, and 400 mM of NaCl. The treated cells were cultured for additional 5 days. Cells grown to the density of 1.5 × 10^7^ cells/ml were centrifuged at 4,000 ×*g* for 10 min; the resulting supernatant was used for luciferase assays. Luciferase activity was measured using the Pierce Gaussia Luciferase Glow Assay Kit (Thermo Fisher), in accordance with the manufacturer’s protocol. Twenty microliters of supernatant and 50 μl of working solution from the kit were mixed and incubated for 10 min. After incubation, luminescence was measured using a microplate reader (Varioskan LUX Multimode Microplate Reader, Thermo Fisher) to determine luciferase activity. Three reactions were prepared for each treatment, and each reaction was measured three times.

## Results

### Identification of NaCl-Induced Genes

The *Chlorella* transformation vector demonstrated targeted nuclear integration by double homologous recombination; thus, genes present in chloroplasts and plastids were excluded. The five genes with the highest fragments per kilobase per million mapped reads (FPKM) values at any time or concentration were selected from the transcriptome analysis (Table 2). Gene 1 encodes a mitochondrial protein; gene 4 encodes an ABC transporter adenosine triphosphate-binding protein; and genes 2, 3, and 5 encode hypothetical proteins. We confirmed the presence of transcripts from the selected genes via quantitative polymerase chain reaction (qPCR) and detected polymerase chain reaction (PCR) products of the expected size for all target genes (Fig. S3). However, we could not identify the promoter regions of genes 3, 4, and 5 because the reference genome sequence of *C. vulgaris* isolate 211/ 11P (GenBank accession number CM041649.1) was incomplete. Furthermore, gene 1 from mitochondria was excluded for nuclear transformation; thus, gene 2 was selected as the final candidate for further analysis.

### qPCR Analysis of Gene 2 under NaCl Stress Conditions

The transcript level of gene 2 under various NaCl conditions was quantified (Fig. 1). The transcript level in untreated wild-type *C. vulgaris* PKVL7422 was used as a reference and compared with the levels in treated samples. The highest transcript level was observed after treatment with 1% (w/v) NaCl for 30 min; this level was 272.9-fold higher than the level in untreated reference cells. However, the transcript level sharply decreased after 30 min. Treatment with 1.5% and 2% NaCl resulted in the highest transcript levels after 120 min and 30 min, respectively, but the increases were only 88.6-fold and 109.9-fold higher than the level in untreated reference cells.

### Analysis of SIP cis-Acting Elements

**Fig. 2:**
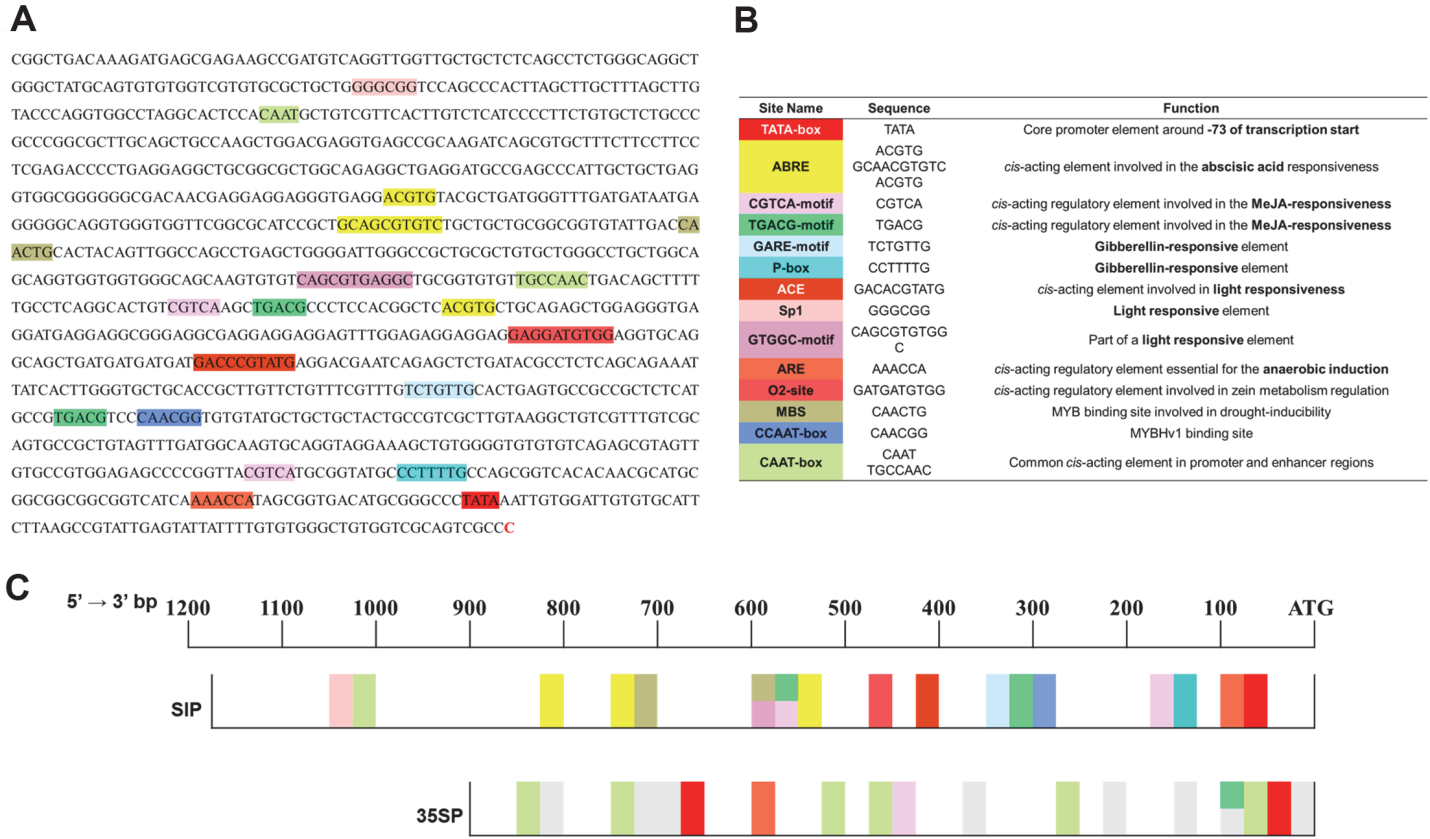
Transcription factor binding and start sites for the SIP. (**A**) Each colored sequence represents a possible transcription factor binding site. Transcription start site (TSS) is shown in red at the end of the sequence. (**B**) Names, sequences, and functions of transcription factors present in the SIP. (**C**) Schematic diagram comparing transcription factor binding sites of the SIP and the CaMV 35S promoter. Light gray boxes indicate binding sites that are only present in the CaMV 35S promoter.

The 1,174-bp-long DNA fragment containing the putative promoter of gene 2 (SIP) was amplified, cloned into the pMD19 vector, and sequenced. The nucleotide sequence of the SIP has been deposited in GenBank under the accession number OQ674258. Analysis of SIP *cis*-acting elements was performed using the PlantCARE database (Fig. 2). Fourteen transcription factor binding elements were identified within the SIP (Figs. 2A and 2B). Comparison of the SIP with the widely used CaMV 35S promoter in the *Chlorella* transformation system revealed five *cis*-acting elements, including a TATA box, an O2 site, a CGTCA motif, a TGACG motif, and a CAAT box (Fig. 2C). Furthermore, we identified stress response-related factors, including ACE (ACGT-containing elements), which responds to light; ABRE (abscisic acid responsive elements), which responds to abscisic acid (ABA); CGTCA and TGACG motifs, which respond to methyl jasmonic acid (MeJA); and a GARE (gibberellin response element) motif and a P-box, which respond to gibberellin (GA). A TATA box was also identified at positions -75 to -73 from the predicted transcription start site.

### Transformation of *C. vulgaris*

**Fig. 3:**
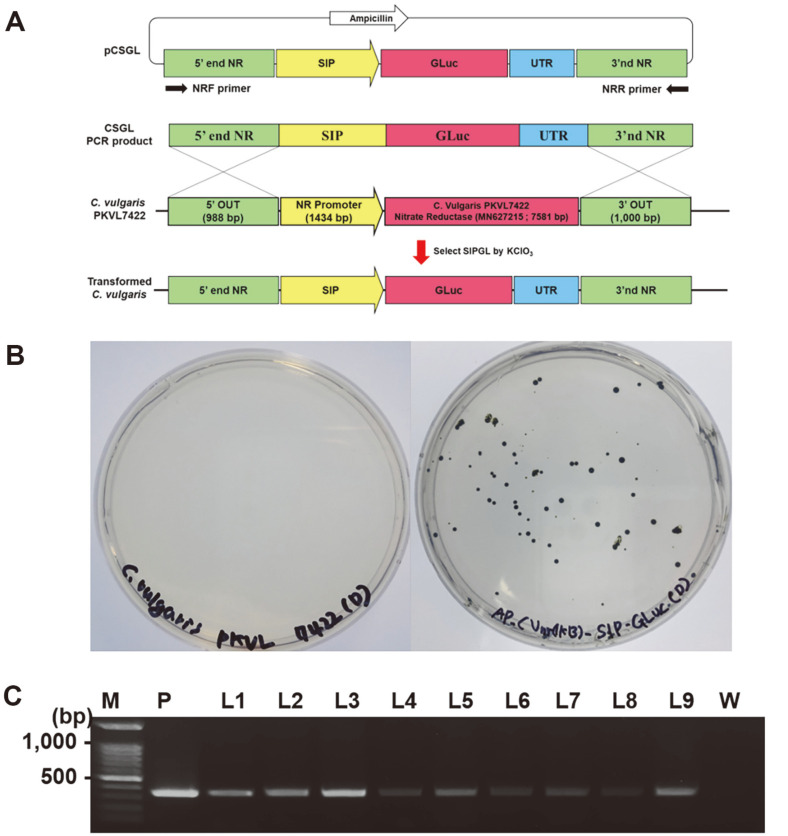
*Chlorella* transformation. (**A**) Schematic overview of *Chlorella* transformation by double homologous recombination and selection of transformants with KClO_3_. (**B**) BGNK plates inoculated with wild-type (left) and transformed *C. vulgaris* PKVL7422 (right). (**C**) PCR results of selected transformed cells showing insert DNA within the genome. Lane M: DM3200 DNA marker (SMOBIO), Lane W: wild-type *C. vulgaris* PKVL7422, Lane P: positive control (plasmid vector), Lanes 1–9: transformed *C. vulgaris*.

*C. vulgaris* PKVL7422 was transformed using an amplified DNA fragment (Fig. 3A) and selected on a BGNK plate. After 2 weeks of incubation under dark conditions, colonies of transformed cells grew on the plate, whereas colonies of wild-type cells did not (Fig. 3B). PCR amplification using the Gaussia luciferase gene (GLuc) primer set (Table 1) confirmed the presence of the corresponding DNA in the genomes of all selected colonies (Fig. 3C).

### Induction of the SIP with Different Stress Factors

**Fig. 4:**
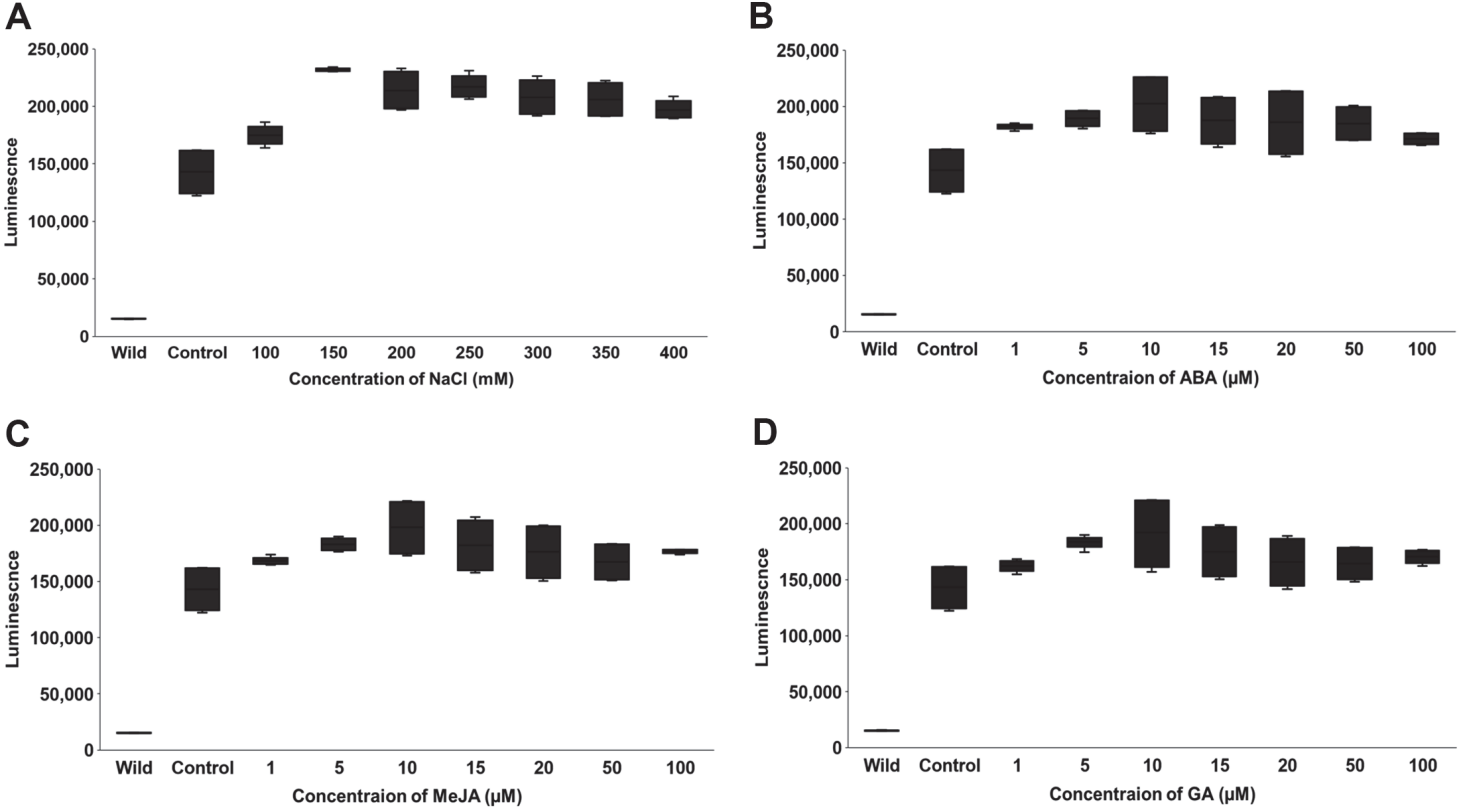
SIP inducibility measured by luciferase activity. Luciferase activity after treatment with 100–400 mM NaCl (**A**), 1–100 μM abscisic acid (**B**), 1–100 μM methyl jasmonic acid (**C**) and 1–100 μM gibberellin (**D**). Wild: wild-type *C. vulgaris* PKVL7422, Control: untreated transformed *C. vulgaris*. Error bars represent standard deviations based on three replicates.

Because the SIP was isolated from a gene that exhibited increased transcription in response to NaCl treatment, transformed *Chlorella* cells were treated with NaCl and their luciferase activities were measured. Transformed cells that were not treated with NaCl used as a control also showed a significant level of luciferase activity. In NaCl treatment, luciferase activity increased up to 150 mM NaCl which was 1.6 times of the control, remained stable up to 350 mM NaCl, and slightly decreased at 400 mM NaCl (Fig. 4A). Furthermore, transformed *Chlorella* exhibited increased luciferase activity when treated with ABA, MeJA, and GA, compared with the untreated control. The highest luciferase activity was observed at 10 μM of all these substances and decreased in higher concentrations, but was higher than that of the control (Figs. 4B-4D).

## Discussion

Salt stress can have a substantial impact on plants, leading to oxidative stress and secondary stresses that can hinder germination and fruiting [29]. Plants have complex transduction pathways that allow them to respond to salt stress via regulation of ion homeostasis, osmotic responses, hormone signaling, cytoskeleton dynamics, and cell wall composition [30]. Freshwater microalgae are also vulnerable to salt stress, which can cause oxidative damage, chlorophyll degradation, and the inhibition of growth and photosynthesis [31]. Although potential mechanisms for microalgal responses to salt stress have been proposed (*e.g.*, Ca^2+^ signaling, calmodulin and calmodulin-dependent kinases, and gamma-aminobutyric acid [GABA]), detailed mechanisms underlying the regulation of gene expression levels have not been elucidated [32].

Although constitutive promoters such as the RbcS2 promoter can be used to produce low-maintenance algal transformants, inducible promoters are preferable because they can be activated under specific environmental conditions and do not have negative effects on the algal cell metabolism and productivity, unlike constitutive overexpression [2]. Promoters that induce gene expression under specific environmental factors are well-characterized, whereas only a few studies on SIPs of microalgae transformation systems exist. For example, Beltran-Aguilar *et al*. [19] showed that the CrGPDH3 promoter of *C. reinhardtii* can be induced by NaCl treatment in transformed *C. reinhardtii*. In a previous study, we confirmed that various endogenous factors were up- or downregulated in *C. vulgaris* PKVL7422 subjected to salt stress [22]. Based on these data, we identified a nuclear gene, gene 2, which displayed a 272.9-fold increase in transcription after treatment with 1% NaCl for 30 min (Fig. 1).

The promoter part of gene 2 (SIP) shares five *cis*-acting elements with the CaMV 35S promoter, which is commonly used for microalgae transformation. The TATA box, a core element in eukaryotic promoters, was identified at position -73 in the SIP (compared with position -47 in the CaMV 35S promoter); there were no other regulatory elements between the TATA box and the transcription start site of the SIP. Eukaryotic TATA boxes usually consist of TATAWAWN and are located approximately 30 to 25 bp before the transcription start site [33]. However, the location of this core motif varies. For example, the TATA box was identified at position -299 of the *β*-carotene ketolase gene (bkt1) promoter in *Haematococcus pluvialis* and was essential for promoter activity [34]. In contrast, 21 TATA boxes and 22 CAAT boxes were identified in the 1.02-kb-long Pt211 promoter of *Phaeodactylum tricornutum* [35].

Sequence analysis of the SIP revealed the presence of ABRE, ACE, ARE (anaerobic responsive element), a CGTCA motif, a GARE motif, a GTGGC motif, a P-box, a sp1 gene, and a TGACG motif (Fig. 2); these elements are involved in response pathways to abiotic stresses such as ABA, light, anaerobic conditions, MeJA, and GA [14, 36, 37]. As predicted, luciferase activity increased in transformed *C. vulgaris* as NaCl concentration increased (Fig. 4A). Although FPKM value of the gene 2 was the highest at 2 h treatment with 3% NaCl as shown in Table 2, treatment of *C. vulgaris* at this concentration resulted in cell death within 2 to 3 days incubation. Total cell mass can affect the yield of recombinant proteins and NaCl concentration up to 400 mM at which transformed *C. vulgaris* can grow was applied to the luciferase assay in this study [39].

Salt stress is a type of abiotic stress that results in cellular dehydration, ionic stress, and oxidative stress associated with osmotic pressure difference between cells and the surrounding medium; it rapidly induces the synthesis of the stress response factors ABA, jasmonic acid (JA), GA, and indole-3-acetic acid [37, 38, 40]. Therefore, these chemicals were applied to the transformed *C. vulgaris*; induction activity was then measured based on luciferase activity, which was linked to the applied concentration of NaCl (Figs. 4B-4D).

The ABRE element of the SIP responded to ABA, which is one of the most important hormones involved in stress response regulation in plants and microalgae [36, 38]. In response to NaCl stress, ABA synthesis is rapidly induced in *Arabidopsis thaliana* [38]. When the ABA level increases, the kinase cascade is activated to function as a secondary signal transducer that mediates gene expression, improving the recognition of stress and the subsequent response [30, 41]. In a previous study, we confirmed the upregulation of genes involved in ABA synthesis in *C. vulgaris* PKVL7422 that had been exposed to salt stress [22].

JA is involved in stress signaling in microalgae; increases the expression levels of carotenoid-related genes; and contributes to growth, development, and metabolism control [37]. SIPs contain the CGTCA and TGACG motifs, which are MeJA-focused regulatory factors. Salt treatment of *C. vulgaris* PKVL7422 increased the RNA levels of genes involved in the synthesis of oxo-phytodienoic acid, a precursor of JA that is converted to MeJA [22, 42]. In the present study, the addition of MeJA to transgenic *Chlorella* led to increased luciferase activity (Fig. 4C).

GA is involved in carbon metabolism in microalgae, where it affects growth and overall metabolism [43]. GA also activates antioxidant enzymes under stress; and it accumulates ABA and glutathione to facilitate stress adaptation [43]. GARE and the P-box identified in the SIP are involved in the response to GA [37]. When transgenic *Chlorella* was treated with GA, it demonstrated enhanced luciferase activity, indicating that GA acts on the SIP (Fig. 4D). Our previous study showed that salt stress enhanced the transcription of genes involved in glutathione synthesis [22].

**Fig. 5:**
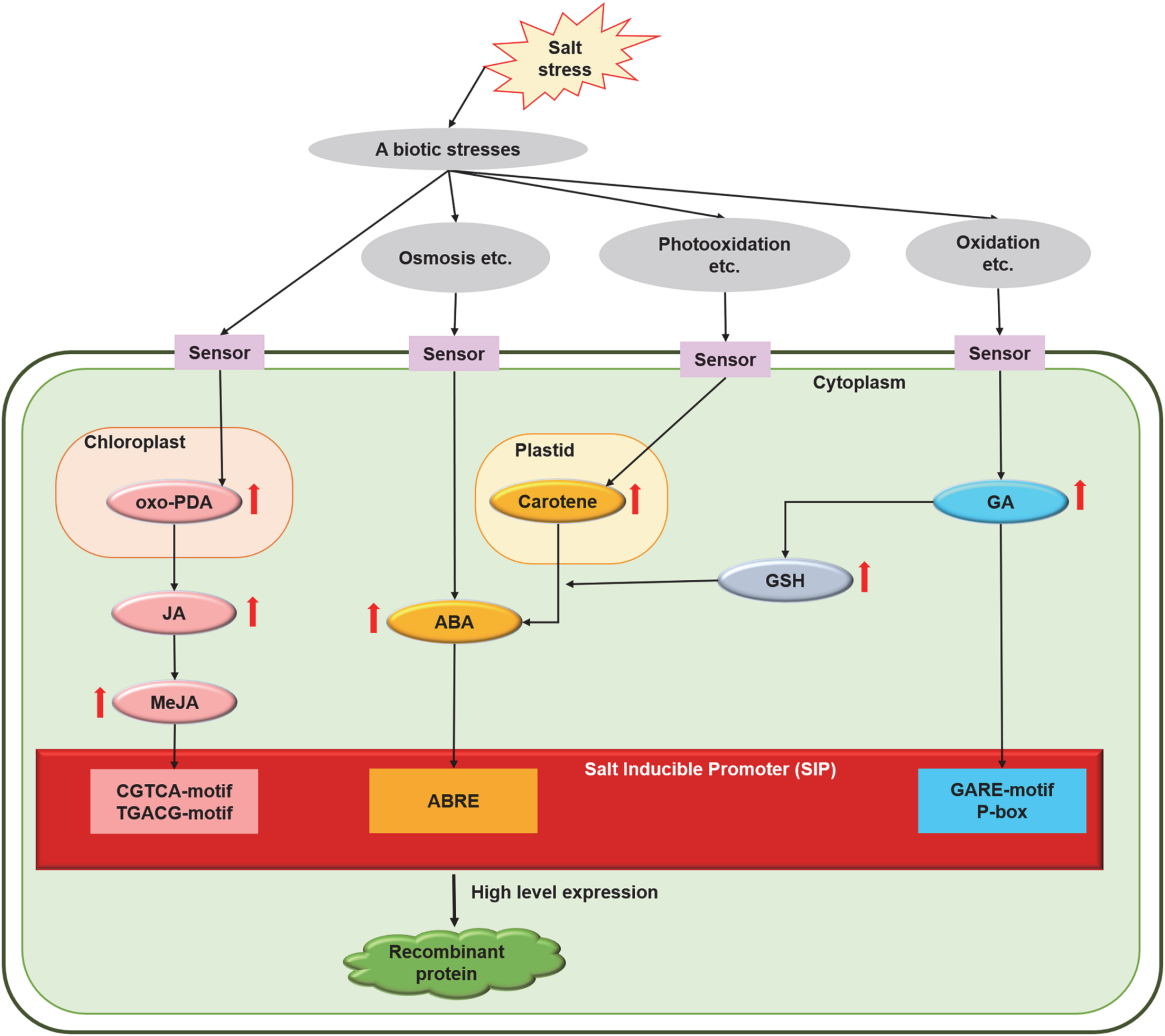
Schematic overview of salt stress response and SIP induction system in *Chlorella vulgaris* PKVL7422. Salt stress transmits an exogenous stress signal into *Chlorella* cells through an unknown transporter. Upregulation of the genes involved in the synthesis of intermediate and final products was confirmed in our previous transcriptome analysis (Abdellaoui *et al*., 2019); SIP induction was confirmed in the present study.

ABA is induced by abiotic stresses such as osmotic pressure, low temperature, and salt stress [44]. Carotene, which is induced by abiotic stresses (*e.g.*, photooxidation and salt stress) is the precursor for ABA [36]. The level of oxo-phytodienoic acid increases in response to abiotic stress, leading to the synthesis of MeJA [46]. GA is induced by salt-related oxidative stress in plants [47], and the GA-induced production of glutathione enhances the synthesis of ABA [48]. Although the pathways active in plants under abiotic stress are well-known, similar pathways in microalgae are not fully understood. Based on the transcriptome analysis performed in our previous study and the promoter activity analysis performed in this study, we propose a possible response pathway to salt stress in *C. vulgaris* PKVL7422, including its activation of the SIP (Fig. 5).

In summary, we isolated the SIP of the freshwater microalgae *C. vulgaris* PKVL7422 by analyzing the transcriptome of salt-treated cells. The isolated SIP was fused to the luciferase gene and transformed into *C. vulgaris* PKVL7422. When the transformant was treated with salt and chemicals that are induced in salt-stressed *C. vulgaris* cells, it demonstrated increased luciferase activity. Although microalgae hold promise as potential bioreactors for the production of valuable recombinant proteins, their lack of highly inducible promoters limits their widespread use as bioreactors. The SIP isolated in this study could help to solve this problem. For example, transformed *C. vulgaris* PKVL7422 could be treated with NaCl to significantly enhance the expression of the recombinant glycoprotein of viral hemorrhagic septicemia virus, which can be utilized for oral vaccine development (Fig. S2).

## Supplemental Materials

Supplementary data for this paper are available on-line only at http://jmb.or.kr.

## Figures and Tables

**Table 1:** Primers used in this study.

Target	Primer name	Sequences	PCR product
Gene1	Gene1_F	5’-AGATTTAGTGGCTTGGGTAGC-3’	123 bp
	Gene1_R	5’-ATATGTGCCGGAGTTGACATAG-3’	
Gene2	Gene2_F	5’-CGCGCACTCTGCCATATAA-3	107 bp
	Gene2_R	5’-GTTGAGCAAATCGTCACCAATC-3’	
Gene3	Gene3_F	5’-CAGTCGGTAGCAGCTTTCTT-3’	90 bp
	Gene3_R	5’-GGGTGCCATCGTAATCATAGT-3’	
Gene4	Gene4_F	5’-CGCGCACTCTGCCATATAA-3’	145 bp
	Gene4_R	5’-GTTGAGCAAATCGTCACCAATC-3’	
Gene5	Gene5_F	5’-CTTCGACTTCCGACGACTTT-3’	121 bp
	Gene5_R	5’-GAGCCAGCATAGTCAGCATAG-3’	
SIP	SIPF	5’-GAATTCGCCTGTCGCTGTTCTGCACGG -3’	1,174 bp
	SIPR	5’-GGATCCGGCGACTGCGACCACAG-3’	
NR	NRF	5’-ATGGACAAGACAGGGTTCGG-3’	5,013 bp
	NRR	5’-AATACAGGCGGAGCCCAAAC-3’	
GLuc	GLucF	5’-CAAGCCGACCGAGAACAAC -3’	350 bp
	GLucR	5’-CCTGCGCGATAAACTGCTC -3’	

**Table 2:** FPKM values of genes selected from transcriptome analysis of NaCl-treated *C. vulgaris* PKVL7422.

	Gene description	170 mM (1%) NaCl 2 h	170 mM (1%) NaCl 4 h	510 mM (3%) NaCl 2 h	510 mM (3%) NaCl 4 h	Gene function
Gene 1	AST08696.1 cytochrome b (mitochondrion)	3.93	9.89	2.84	9.34	Mitochondrial electron transport, ubiquinol to cytochrome c
Gene 2	XP_005848099.1 hypothetical protein	37.72	25.64	74.22	63.89	Nucleotide binding
Gene 3	XP_005845324.1 hypothetical protein	31.44	18.18	-1.89	-2.29	Nucleotide binding
Gene 4	PRW44363.1 ABC transporter ATP-binding	12.57	9.98	3.39	2.07	Integral component of membrane
Gene 5	XP_005843025.1 hypothetical protein	19.42	8.74	66.95	13.14	Membrane
