# Supplementary material for: Isolation and Characterization of a Salt Inducible Promoter from Chlorella vulgaris PKVL7422
Source: J Microbiol Biotechnol. 2023 Apr 28;33(7):955–63. doi: 10.4014/jmb.2304.04005 (PMC10394332; doi:10.4014/jmb.2304.04005)
Supplement: Supplementary file 1 [file jmb-33-7-955-supple.pdf]

J. Microbiol. Biotechnol.
https://doi.org/10.4014/jmb.2304.04005

Supplementary Figures

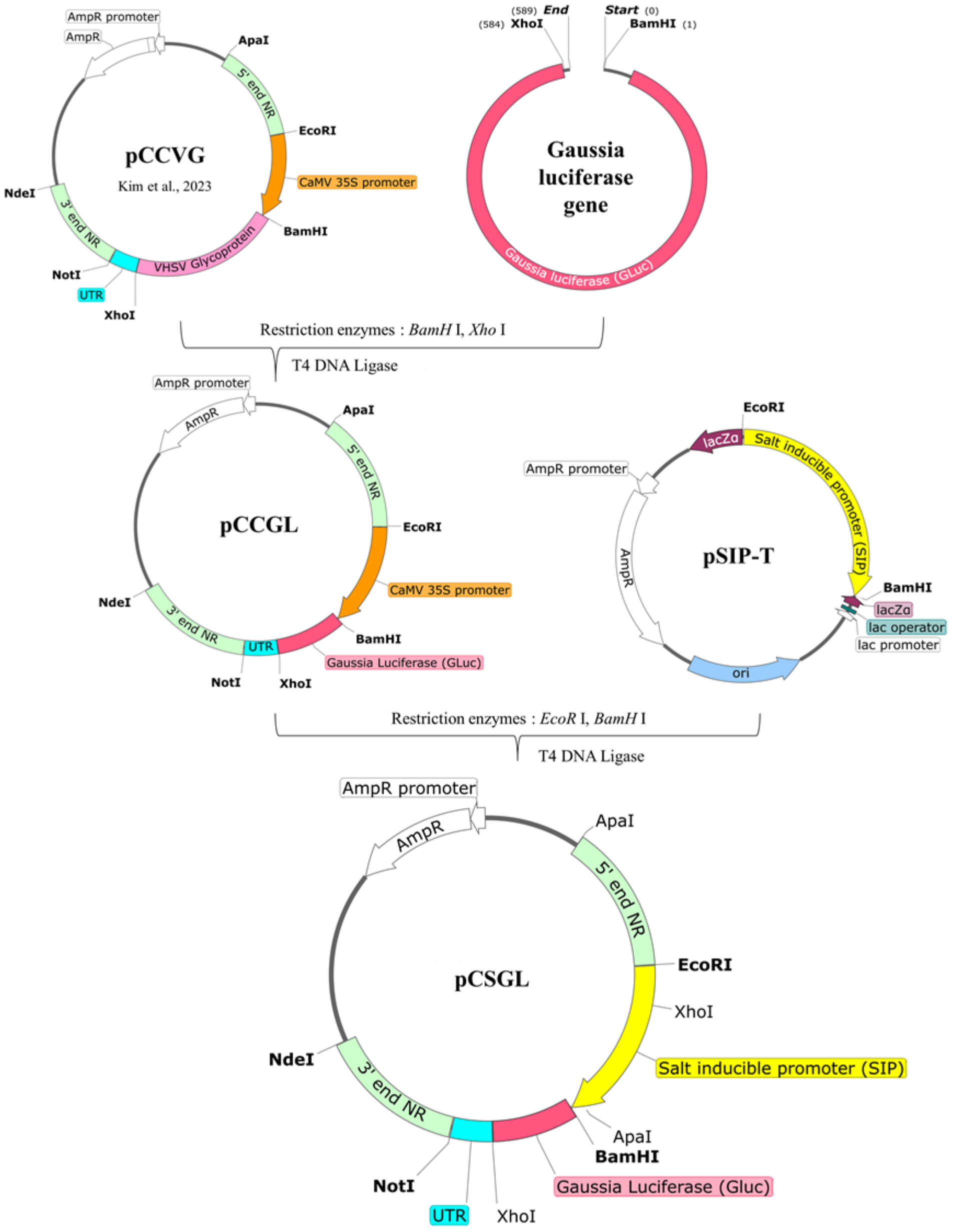

Supplementary Figure 1.

Schematic overview of *Chlorella* transformation vector construction. Three vector maps for transformation into *C. vulgaris* PKVL7422 are shown. Detailed methods are described in the text. The DNA fragment between the *Apa*I and *Nde*I sites in the pCCVG vector was

amplified by PCR and used for transformation.

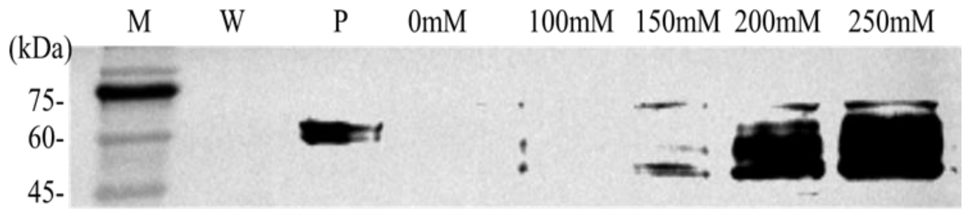

Supplementary Figure 2.

Enhancement of recombinant protein expression under SIP control via salt treatment of transformed *Chlorella*. The luciferase gene in the pCSGL vector of Figure S1 was replaced with the glycoprotein (G) gene of viral hemorrhagic septicemia virus (VHSV), a fish-infecting virus, then transformed into *C. vulgaris* PKVL7422. After treatment of the transformed cells with 100–250 mM NaCl for 5 days, protein expression was analyzed by Western blotting with antibodies against the G protein. Lane M: PM2700 protein marker (SMOBIO), Lane P: positive control (purified VHSV), Lane W: total protein from wild-type *C. vulgaris* PKVL7422, Lanes 0–250 mM: total protein from transformed *C. vulgaris* that had been treated with the indicated concentrations of NaCl.

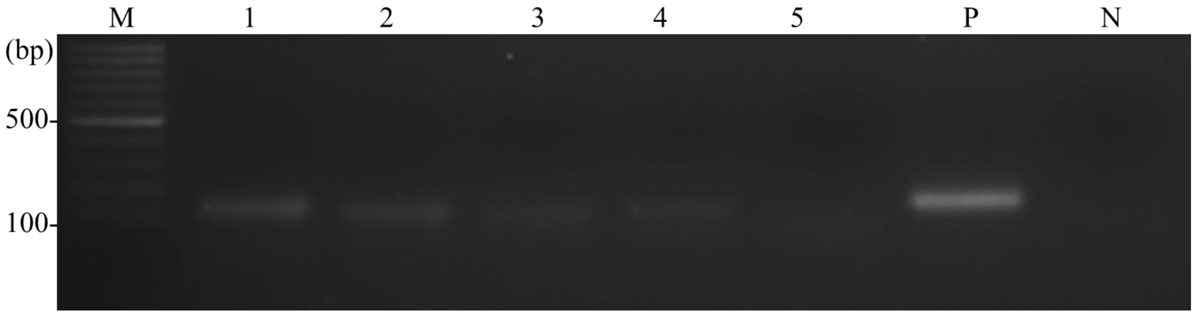

Supplementary Figure 3. The result of RT-PCR confirmation of 5 genes with the most unigenes up-regulated under salt stress. Lane M; DM 3200, DNA marker. Lane 1-5; Gene 1-5. Lane P; Positive control (18s rRNA). Lane N; Negative control.
